# Adaptation of the Gabby conversational agent system to improve the sexual and reproductive health of young women in Lesotho

**DOI:** 10.3389/fdgth.2023.1224429

**Published:** 2023-10-04

**Authors:** Elizabeth Nkabane-Nkholongo, Mathildah Mokgatle, Timothy Bickmore, Clevanne Julce, Brian W. Jack

**Affiliations:** ^1^ School of Public Health, Sefako Makgatho University of Health Sciences, Pretoria, South Africa; ^2^ Khoury College of Computer Sciences, Northeastern University, Boston, MA, United States; ^3^ Chobanian & Avedisian School of Medicine, Boston University, Boston, MA, United States

**Keywords:** preconception care, conversational agent technology, women’s health education, mHealth adaptation, health education in global health

## Abstract

**Introduction:**

Young women from the low-middle-income country of Lesotho in southern Africa frequently report limited knowledge regarding sexual and reproductive health issues and engage in risky sexual behaviors. The purpose of this study is to describe the adaptation of an evidence-based conversational agent system for implementation in Lesotho and provide qualitative data pertaining to the success of the said adaptation.

**Methods:**

An embodied conversational agent system used to provide preconception health advice in the United States was clinically and culturally adapted for use in the rural country of Lesotho in southern Africa. Inputs from potential end users, health leaders, and district nurses guided the adaptations. Focus group discussions with young women aged 18–28 years who had used the newly adapted system renamed “Nthabi” for 3–4 weeks and key informant interviews with Ministry of Health leadership were conducted to explore their views of the acceptability of the said adaptation. Data were analyzed using NVivo software, and a thematic content analysis approach was employed in the study.

**Results:**

A total of 33 women aged 18–28 years used Nthabi for 3–4 weeks; eight (24.2%) of them were able to download and use the app on their mobile phones and 25 (75.8%) of them used the app on a tablet provided to them. Focus group participants (*n* = 33) reported that adaptations were culturally appropriate and provided relevant clinical information. The participants emphasized that the physical characteristics, personal and non-verbal behaviors, utilization of Sesotho words and idioms, and sensitively delivered clinical content were culturally appropriate for Lesotho. The key informants from the Ministry leadership (*n* = 10) agreed that the adaptation was successful, and that the system holds great potential to improve the delivery of health education in Lesotho. Both groups suggested modifications, such as using the local language and adapting Nthabi for use by boys and young men.

**Conclusions:**

Clinically tailored, culturally sensitive, and trustworthy content provided by Nthabi has the potential to improve accessibility of sexual and reproductive health information to young women in the low-middle-income country of Lesotho.

## 1. Introduction

Adolescents face many sexual and reproductive health problems worldwide, including unplanned pregnancy, sexually transmitted infections, and human immunodeficiency virus (HIV) infections (1). Adolescents account for 42% of new HIV infections globally, and four in five young people with HIV live in sub-Saharan Africa (2). Lesotho has the second-highest HIV prevalence in the world, accounting for 22.7%, and one of the highest HIV incidence rates among adolescent girls and young women accounting for 0.33% (3). The maternal mortality rate in Lesotho of 544/100,000 live births is the second highest in Southern African Development Community countries (4).

In Lesotho, adolescent girls and young women frequently report limited knowledge in sexual and reproductive health issues and engage in risky sexual behaviors. They are at increased risk of early sexual debut (14 years), unprotected sex, and multiple sexual partners, exposing them at risk of acquiring sexually transmitted infections, including HIV and unintended pregnancy (5). Providing sexual and reproductive health education to young women is challenging in this rural, mountainous country (6, 7) and where health professionals face constraints related to time and resources.

New tools to deliver health education are needed. In Lesotho, 94% of people aged 18–29 years use smartphones, and 3G data coverage is available in almost 90% of the country (8, 9). The high penetration of mobile technologies provides an opportunity to explore the use of new tools delivered on mobile phones as an alternative to the traditional face-to-face provision of health education (10).

Embodied conversational agents (ECAs) are computer-based animated characters designed to simulate face-to-face human interactions. They are an effective medium to educate patients with limited health or computer literacy, as the human–computer interface relies only minimally on text comprehension and prioritizes conversation making it more accessible to patients with limited literacy skills (11, 12). Non-verbal conversational behaviors, such as hand gestures that convey specific information through pointing, shape, or motion, are channels for conveying information on semantic content that enhances message comprehension (11).

**Figure 1:**
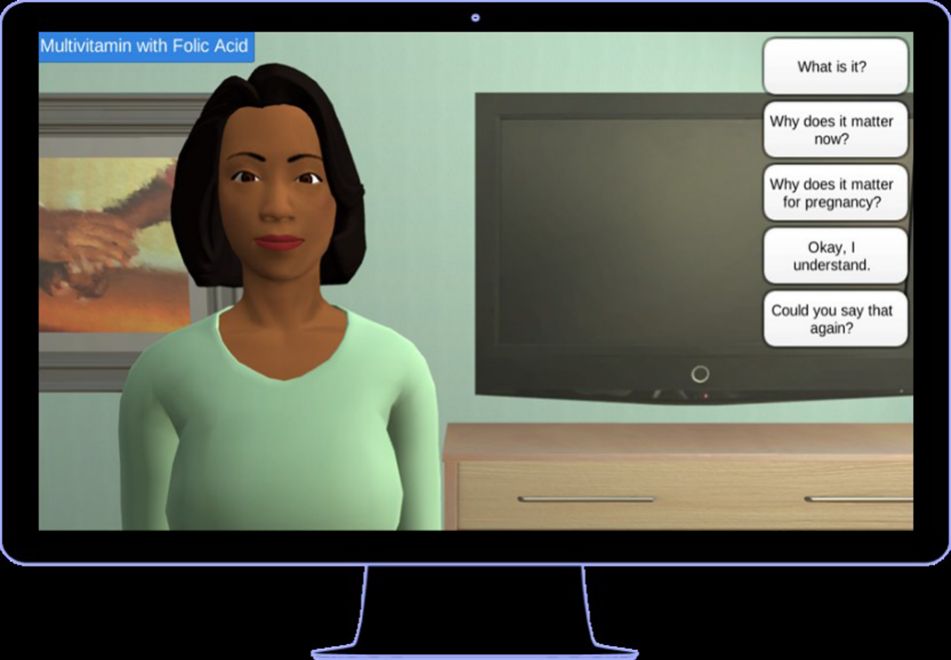
Gabby was developed in the United States to deliver health education that women could access on desktop computers.

An ECA called Gabby was designed to deliver sexual and reproductive health information to African American women of reproductive age in the United States that demonstrated significant improvement in addressing reproductive health risks (see Figure 1) (13, 14).

This paper describes the process used to adapt Gabby for use in Lesotho, and provides qualitative data regarding the success of the adaptation, collected from potential end users and Ministry of Health (MOH) leadership.

## 2. Methods

### 2.1. Conceptual model

The PEN-3 cultural utility model guided the adaptations, which provides valuable guidelines for ensuring a culture-specific intervention by identifying and organizing a community's culture in the planning processes (15–17). The model includes the cultural identity domain (person, extended family, and neighborhood), the relationship and expectation domain (perceptions, enablers, and nurturers), and the cultural empowerment domain (positive, existential, and negative) (18–20).

Adaptations were also guided by the heuristic framework for cultural adaptations of Barrera et al. (21), which includes information gathering, preliminary adaptation design, preliminary adaptation tests, adaptation refinement, and cultural adaptation trial.

### 2.2. Information gathering for adaptation

To understand the adaptations needed, the Northeastern University team leader visited Lesotho in January 2020. Meetings were held with MOH managers for adolescent health, HIV, family planning, and nursing to elicit recommendations with regard to potential adaptations. Meetings were also conducted with district nurses and with young women seeking healthcare services.

The meetings elicited details with regard to how Basotho use their hands, facial gestures, and body language in conversation and ideas regarding the use of Basotho language and cultural references, such as idioms, that could be incorporated into the new system. The *persona* of the new character was explored to identify those that would lead to greater trust in the health information being delivered. Interviews highlighted the importance of promoting engagement with the new system. Images of Basotho women were used to create the character. Decisions were made regarding the character's appearance (e.g., hair and clothing), behavior (speech pattern), nationality, sex, age, occupation, and name.

The MOH officials were asked to recommend topics that they believed to be critical for improving sexual and reproductive health for adolescents and young women. The topics agreed upon for inclusion in the system were family planning, HIV and AIDS, tuberculosis, healthy eating, and folic acid supplementation. Boston University and the Lesotho team then used the Lesotho national guidelines on these topics to prepare the dialogs to be delivered by Nthabi.

Technical adaptations were required to deploy the prototype Gabby interface (designed to be displayed on a computer screen) to now be displayed on smartphone screens. The new Nthabi system interface was designed to display only the face and response options of the characters.

The development team sourced Lesotho mobile phones to gain information regarding the specifications penetration of devices. To increase the accessibility and use of the system, a decision was made to ensure that the app can be completely downloaded into the user's mobile phone, thereby enabling use even outside of WiFi-enabled environments. Data pertaining to user usage and the content discussed would be downloaded when the user is next in a WiFi-enabled environment.

### 2.3. Recruitment and enrollment of participants

The participants were recruited to use Nthabi in accordance with predetermined eligibility criteria: aged 18–28, owned a smartphone, spoke English, and lived in the Leribe or Berea districts of Lesotho. A purposive sampling technique was employed to recruit participants when they accessed services at the adolescent and maternal and child services at district hospitals. The Nthabi app was downloaded onto the participants’ mobile phones after they provided informed consent. The participants who were unable to download the app were provided with an internet-accessible tablet device. All participants were asked to use the system daily for 4 weeks.

### 2.4. Focus groups of participants using Nthabi

Young women who used the Nthabi system were contacted to arrange for their participation in focus groups to elicit their perceptions of the system. Four focus groups were conducted between July and August 2022. The groups were facilitated by the first author using an interview guide with open-ended questions designed to explore the cultural and clinical adaptation, ease of use, problems encountered, willingness to continue use, and possible future use. Focus group participants were provided a stipend of 50 Maloti (∼US $3).

### 2.5. Key informant interviews with MOH leaders

Purposive sampling was employed to recruit MOH program managers and adolescent health nurses to participate in key informant interviews. They were asked to review the content of a video shared on WhatsApp of the key parts of the Nthabi interactions for 3–5 days. In June and July 2022, interviews to elicit their perceptions were conducted using an interview guide with open-ended questions used to explore cultural and clinical adaptation and perceptions of use to deliver health education in the country. These participants did not receive a stipend.

## 3. Data analysis

Data analysis was headed by the Sefako Makgatho University team. All interviews and focus groups were approximately 1 h and were audio recorded. The study team members transcribed semi-verbatim all audio recordings into Microsoft® Word and checked for accuracy, and extraneous sounds, remarks, or repetitions were omitted in the transcription. In addition, words were added, as appropriate, for clarity. They were not returned to participants for correction. Before analysis, all identifying data were removed, and Sesotho words were translated into English.

All qualitative data were imported into QSR International's NVIVO v12 software for coding and analysis. We conducted a thematic analysis using a combined inductive and deductive approach to coding, starting with broad codes from the interview guide and allowing room for new codes to emerge. Given the heterogeneity of respondent demographics, we coded all interviews instead of stopping when saturation was researched within particular thematic areas. A coding tree was produced that contained emergent categories of barriers and facilitators and re-coded the data. The core major and minor themes were determined through iterative inputs from authors on the resultant thematic map (22).

The demographic data were collected from the participants at enrollment and were presented as counts, frequencies, and means. All information was stored on encrypted tablets.

## 4. Results

### 4.1. Initial consultative meetings

Initial consultative meetings were held with eight MOH directors or program managers, four district nurses who were aged 25–50 years, and nine women aged 18–28 years.

**Figure 2:**
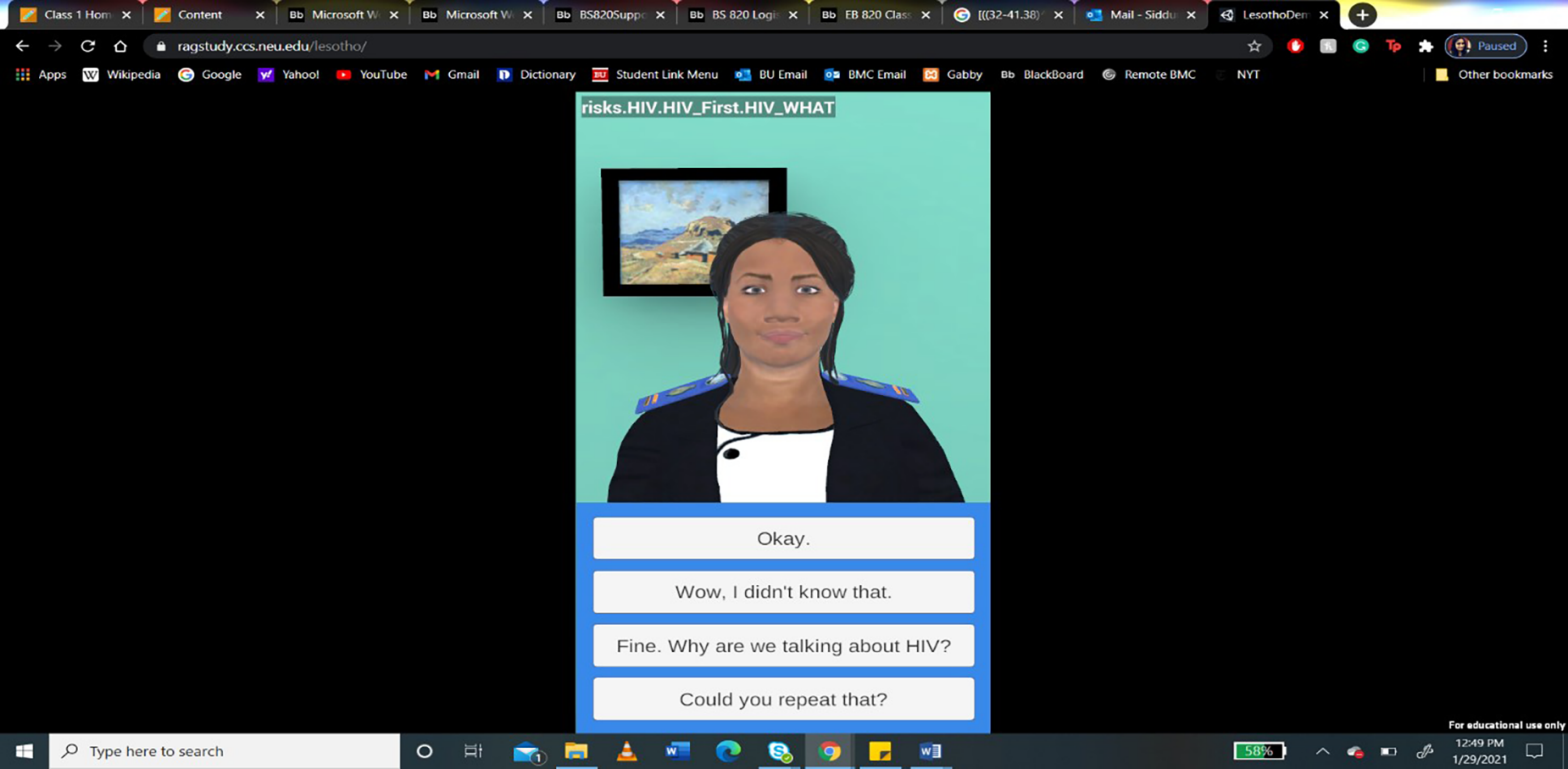
Lesotho version of Gabby (Nthabi) for use on mobile phones.

Based on these discussions, it was recommended that the Lesotho version of Gabby would be a young female nurse named “Nthabi”, wearing a Lesotho nurse's uniform and using Sesotho words and idioms. Her hairstyle (braids), complexion (medium, similar to the local population), use of gestures (calm and gentle), and mannerisms (a humble professional with a sense of humor) would be relatable to young women in Lesotho (see Figure 2). English was chosen as the preferred language for Nthabi, since a Sesotho speech synthesizer was unavailable.

To promote engagement, a professional Mosotho woman artist and storyteller was engaged to write 60 daily installments of a serial story, each ending with a cliff-hanger that could motivate users to use the app daily. Such serialized stories are popular in Lesotho.

### 4.2. Description of participants who used Nthabi

A total of 41 participants who met the eligibility criteria were recruited to use the new app. After giving consent, the participants were assisted to download the Nthabi app onto their mobile phones. Of the 41 eligible participants, 16 (39%) participants were able to download the app onto their phones. If the app could not be downloaded, the participants were loaned a Lenovo© Android 11 OS platform tablet to use. Overall, eight out of 16 (50%) participants who were able to download Nthabi app on their mobile phones used the app consistently. The other eight encountered challenges related to phone memory and freezing of the phone, which leads them to uninstall the app. In total, 25 participants used the Lenovo tablets provided by the research team and eight participants on their phones (total 33).

### 4.3. Focus group discussions and key informant interviews

All 33 participants who used Nthabi participated in focus groups. The mean age of the participants was 23 years, and 27 (82%) of them were single. All had completed high school or above, and 22 (76%) were unemployed (Table 1). All the 10 health leaders who participated in the key informant interviews (mean age of 37.5 years) had received tertiary education and were employed (Table 1).

**Table 1:** Socio-demographic and usage data of key informants and focus group participants.

		Key informants *n* = 10, *N* (%)	Focus group participants *n* = 33, *N* (%)
Age (years)	18–28	3 (30)	33 (100)
	28–38	2 (20)	—
	38–48	3 (30)	—
	49+	2 (20)	—
Marital status	Married	6 (60)	6 (18)
	Single	4 (40)	27 (82)
Education	Tertiary	10 (100)	13 (40)
	High school	—	20 (60)
Employment	Employed	10 (100)	11 (33)
	Unemployed	—	22 (67)
Device used to interact with Nthabi	Mobile phones	—	8 (24.2)
	Tablets	—	25 (75.8)
	Video recording	10	—

*N,* number of participants; %, percentage.

Table 2 summarizes the five themes and subthemes that emerged from the key informant interviews and focus group discussions.

**Table 2:** Summary of themes and subthemes from data reported by the participants of focus groups and key informant interviews.

Themes	Subthemes
1. Appearance and mannerisms	- Physical appearance of Nthabi, including dress, the image that resembles the community of end users, hairstyle, and gestures
2. Acceptability of language used	- Culturally sensitive and acceptable use of language, especially about potentially embarrassing subjects - Non-judgmental
3. Accessibility, relevance, and engagement	- Mobile phones make the information easily accessible and convenient - Nthabi is patient and I did not feel rushed - The serializes stories were engaging
4. Relevance of health content	- Relevance of health education content discussed - Gained knowledge on different empowering health topics and on making good choices

## 5. Theme 1: appearance and mannerisms

The participants described Nthabi as a Mosotho (e.g., singular Basotho) nurse who is friendly, wears her uniform neatly, and provides relevant health education to young women who do not have access to sexual and reproductive health content due to cultural and health provider barriers, such as judgmental attitudes and lack of confidentiality. The participants described Nthabi as relatable to Basotho young women.

### 5.1. Dress code

Both end users and MOH interview participants approved of the Nthabi character being dressed as a nurse. Wearing a nurse’s uniform implies that the information shared by Nthabi is from a reliable source.

*I think she looks good, like the fact that she is a nurse, as this gives young people an assurance that the information that she is providing is credible* (Key Informant 7, 43 years).

### 5.2. Complexion and skin tone

Most participants agreed that Nthabi's skin tone and complexion were relatable to Basotho young women:

*The skin tone indeed is relatable to Basotho. Immediately you see her you definitely can say that is a Mosotho woman* (Key Informant 10, 49 years).

### 5.3. Hairstyle

Most participants liked Nthabi's hairstyle and said that it resembled hairstyles of young Basotho women, although there were suggestions to use other styles.

*I would prefer that the hair be short African hair … plaited in a way that is common in the country, it can be an essence, just a simple thing just to show that she is an African* (Key Informant 8, aged 35 years).

### 5.4. Gestures

The gestures she used in conversation, such as using her hands, facial expressions, and the humility gesture, were viewed positively. End users and key informants agreed that Nthabi used her hands properly, and her facial expressions and the humble character that she portrayed in conversation were culturally appropriate and relatable to young Basotho women.

*I was also surprised with the way the application used gestures, this is a commendable innovation. Truly, she is relatable to Basotho young women* (Key Informant 9, 47 years).

## 6. Theme 2: acceptability of language used

Both end users and key informants appreciated that Nthabi presented information in a tone that is not offensive to the Basotho culture.

*Yes, we can listen to Nthabi with our parents because she chooses her words well and that shows she is culturally sensitive. As Basotho girls, there are some words that cannot be used publicly, but Nthabi seems to know that as well* (Focus group 3, participant 6, 24 years).

*Nthabi is able to provide information about everything even those that a parent or elderly person isn’t comfortable talking about because they are embarrassing* (Focus group 4, participant 4, 21 years).

The participants also believed that they would be comfortable sharing their healthcare needs freely with Nthabi, rather than with healthcare providers because Nthabi is non-judgmental, and they often feel judged by nurses.

*I found Nthabi to be non-judgemental. For instance, say I am 18 years old and I am pregnant, there are certain things I will be told [about] how much I have been dating, etc. This results is feeling discriminated and being uncomfortable to visit the facility again … Nthabi, on the other hand, is open* (Focus group 3, participant 5, 26 years).

## 7. Theme 3: accessibility, relevance, and engagement

Users applauded the Nthabi app since they could access it at times convenient to them rather than relying on having to go to the health center or the hospital. End users said the information provided was important, and they did not feel rushed when talking to Nthabi.

*It was interesting to have a nurse in one's pocket, who is accessible anytime you want to reach out, rather than having to go through long queues at the health facilities to get health information* (Focus group 1, participant 7, 22 years).

*Nthabi is able to ask whether you would like to continue talking to her or if it is enough for the day, meaning she has time for us, not rushing like Nurses* (Focus group 3, participant 5, 26 years).

The participants also reported that the serialized local stories motivated them to use the system every day.

*The story about Thabo and Mpho made [me] more excited. I will always want to know what happens next with Mpho* (Focus group 3, participant 5, 26 years).

## 8. Theme 4: relevance of health content

End users and key informants reported that the five health topics covered by Nthabi are relevant in Lesotho.

*I learned so much about family planning. I did not know about additional methods that I learned from Nthabi* (Focus group 1, participant 3, 21 years).

## 9. Theme 5: suggested modification

End users and key informants suggested several recommendations for additional content including HIV pre-exposure prophylaxis (PrEP), sexual reproduction, teen pregnancy, cervical cancer, and sexually transmitted infections.

*It is very important to actually mention something about cervical cancer, because we know our adolescents actually start to have their sexual debut even before they reach mature age* (Key informant 8, 35 years).

The majority of participants suggested that Nthabi should use the local language, Sesotho, so that information can reach the non-English speaking population. There were also suggestions with regard to her accent.

*Please consider translating all this information to Sesotho, so that young people can have language options* (Key informant 9, 47 years).

*Nthabi's accent should also be rectified because she rolls her tongue a lot and one needs headsets in order to hear other words properly. She isn’t audible enough when you play her on speakerphone* (Focus group 3, participant 3, 23 years).

Finally, the participants suggested that Nthabi could be adapted further for use by boys and young men.

*I also thought the information can be appropriate for boys, please consider an application for boys as well* (Key informant 9, 47 years).

## 10. Discussion

This study found that young women and MOH key informants in the low-middle-income country of Lesotho considered the cultural and clinical adaptation of an evidence-based embodied conversational agent system to have been successful. The clinically tailored, culturally sensitive, and trustworthy content of the Nthabi system has the potential to improve accessibility of sexual and reproductive health information in the rural, mountainous country of Lesotho in southern Africa.

Lesotho faces significant challenges in terms of its healthcare workforce capacity and the effective dissemination of health education (23). There is an urgent need to develop ways to provide trustworthy information regarding sexual and reproductive health, including family planning and condom use to reduce sexually transmitted infections, HIV, unplanned pregnancy, and unsafe abortion in Lesotho.

New technologies are now available to provide evidence-based health education to remote settings with fidelity. Conversational agent technology interactions have been shown to be an effective medium to deliver health education in a variety of topic areas, including to users with limited health literacy (24, 25). Nthabi provides a new opportunity to deliver health education, possibly as an alternative to the traditional face-to-face provision of health education (26, 27).

The study highlights the importance of adaptations of new technologies that represent the unique way of life, behaviors, beliefs, values, and symbols in the Basotho context. Once interactions are defined in relation to appropriate cultural cues, such as language, appearance, gestures, and humility, there is a higher probability of acceptability and usability (28). Substantial testing regarding Nthabi's physical appearance, age, and name was undertaken by the research teams (29).

The study demonstrates that involving stakeholders in the adaptation process can increase the acceptability of systems such as Nthabi. Community participation allowed the system to take on characteristics of the local environment in which it was developed (15, 30). The adaptations considered the language, cultural appropriateness, and context in such a way that is compatible with cultural patterns, meanings, and values of young women (21). This involvement led to incorporation of culturally persuasive features (e.g., physical characteristics, profession, use of Sesotho idioms, storytelling) and addressed issues of potential misfit among the technological, human, and contextual factors. In this way, our findings align with the findings of other studies that utilized the PEN-3 cultural utility model that promoted acceptability (31).

Nthabi's *persona* as a nurse, as well as the incorporation of storytelling as a persuasive feature to promote engagement, was a new innovation in the Nthabi adaptation. These characteristics were not features of the American Gabby system; however, these were recommended in Lesotho as a way to provide assurance that the health education she delivers is reliable and to promote its utilization. These stories of engagement appear to be important in enhancing user experience and encouraging long-term usage, as indicated by the majority of research findings (32).

The MOH leaders who were involved recognized the potential of this technology to provide widely scalable health education, including in population health efforts at the district or national level. Increased awareness among government officials could lead to further research, development, and facilitation of implementation assistance in the country. Focus group and interview participants expressed that expanding sexual and reproductive health education for boys should be considered in the future.

In an Australian adaptation, Gabby's ability to be sensitive to different cultures and languages seemed to be more important than her physical appearance and accent (33). In Lesotho, the participants had differing views on Nthabi's accent, as some felt it needed to be modified to sound more like the local accent. Furthermore, it was recommended that she uses the local language, which shows the necessity of harnessing local meanings and contextual factors in adapting evidence-based interventions (34).

The participants in Lesotho emphasized the importance of physical characteristics, character, hand gestures, Sesotho name, and using Sesotho words and idioms. This finding complements the findings of the study conducted by Fendt-Newlin et al. (30), which emphasized that it must be acknowledged that straightforward extrapolation of the existing evidence base is not always appropriate.

Despite the high penetration of smartphones among young women in Lesotho, only eight of the 33 participants succeeded in downloading the app onto their mobile phones, primarily due to limited memory on many phones and the unavailability of the app in the Google Play Store, especially for the latest Huawei phones, such as the P50 Pro. The research team opted to offer full downloadability of the app onto mobile phones, thereby allowing its use in non-WiFi environments in order to increase the accessibility and use of the system. The results showed that possible improved accessibility is balanced by the size of the app (particularly the speech synthesizer), which limits the downloading of the app to phones. The app could be accessed more easily if it were on the Cloud, and the participants were able to access the Internet; however, this would limit accessibility. Balancing offline accessibility and wide usage of mobile phones needs to be addressed and should be an important consideration in future developments.

This study is limited in that it was an exploratory qualitative study of users’ perceptions. The findings should be confirmed using quantitative methods, such as a survey instrument and improved knowledge acquisition. While the results reflect successful adaptation, it is not known whether the adapted Nthabi system will improve users’ health knowledge or change attitudes and behaviors. Ultimately, systems such as Nthabi will need to be tested to measure impact on important clinical outcomes.

## 11. Conclusion

The culturally adapted Nthabi character and trustworthy relevant content has the potential in enhancing the accessibility of sexual and reproductive health information for young women in Lesotho. Furthermore, this approach has the potential to serve as an alternative to traditional face-to-face health education methods. Suggested modifications include adopting the local language and accent and adapting Nthabi for use by boys and young men. Balancing the size of the app and accessibility in non-WiFi-enabled environments is needed in future deployments.

## Data Availability

The raw data supporting the conclusions of this article will be made available by the authors, without undue reservation.
